# Supplementary material for: Associations between Intake of Dietary Sugars and Diet Quality: A Systematic Review of Recent Literature
Source: Nutrients. 2024 May 21;16(11):1549. doi: 10.3390/nu16111549 (PMC11174080; doi:10.3390/nu16111549)
Supplement: Supplementary file 1 [file nutrients-16-01549-s001.zip › nutrients-3005153-supplementary.pdf]

Associations between intake of dietary sugars and diet quality: A systematic review of recent literature, by Cara et al., 2024

Supplemental materials

Search Strategy
Database: Ovid MEDLINE(R) <1946 to October Week 4 2022>
----
1 sweetening agent*.mp. or Sweetening Agents/ (7493)
2 nutritive sweetener*.mp. or exp Nutritive Sweeteners/ (5211)
3 disaccharide*.mp. or Disaccharides/ (17669)
4 Sucrose/ or sucrose.mp. (76539)
5 dietary sucrose.mp. or exp Dietary Sucrose/ (4672)
6 monosaccharide*.mp. or Monosaccharides/ (25187)
7 fructose.mp. or Fructose/ (43845)
8 (Levulose or Fleboplast Levulosa or Levulosa, Fleboplast or Levulosa Grifols or Levulosado Vitulia or Levulosa Braun or Levulosado Braun or Levulosa Ife or Levulosado Bieffe Medit or Apir Levulosa or Levulosa, Apir or Levulosa Mein or Plast Apyr Levulosa Mein or Levulosa Ibys or Levulosa Baxter or Levulosa).mp. (130)
9 high fructose corn syrup.mp. or exp High Fructose Corn Syrup/ (578)
10 dietary sugar*.mp. or exp Dietary Sugars/ (6044)
11 honey.mp. or exp Honey/ (11556)
12 molasses.mp. or exp Molasses/ (2200)
13 (sugar* adj2 (table or cane or beet* or fruit or white or brown or turbinado or muscovado or free-flowing or liquid or invert or maple or date or raw or refine* or powder* or confection* or baker* or granulat* or superfine or coarse or sanding or total or simple or free or extrinsic or added or intake*)).mp. (15204)
14 (sugarcane or jaggery or HFCS or attar or acetomel or chashni or cheong or cherry smash or cordial or dilute or falernum or grenadine or kuromitsu or mizuame or pekmez or squash or treacle or vincotto or yeot).mp. (21398)
15 syrup*.mp. (5792)
16 sweeten*.mp. (14731)
17 (sweet* adj2 sorghum).mp. (271)
18 (juice adj2 (concentrate* or dilut* or cane)).mp. (679)
19 (diet* adj2 (sugar* or sucrose* or saccharose*)).mp. (8894)
20 (non-milk extrinsic sugars or NMES).mp. (1276)
21 or/1-20 (211570)
22 (dietary fiber* or dietary fibre*).tw. or exp Dietary Fiber/ (26529)
23 ((fibre* or fiber*) adj2 (intake or dietary or functional or added or pea or potato or rye or soluble corn or sugar cane or sugar beet or sugarbeet)).tw. (13329)
24 (Alginates or Alphacyclodextrin or Alpha-cyclodextrin or Arabinoxylan or Arabinoxylan-oligosaccharides or AXOS).tw. (3237)
25 Aleurone.tw. (916)
26 (bran adj2 (barley or corn or oat or rice or rye or wheat)).tw. (4825)
27 (Barley grain or Beta-glucans).tw. (2058)
28 (Carrageenans or Cellulose or Chitin or Chitosan).tw. (92353)

29 (resistant adj2 (maltodextrin or starch or dextrin*)).tw. (1950)
30 (Fructan* or Fructooligosaccharide* or Fructo-oligosaccharide* or short chain fructo-oligosaccharide or short chain fructo oligosaccharide).tw. (2989)
31 (Galactooligosaccharide* or Gum* or Galactomannan or Arabinogalactan).tw. (21476)
32 (hemicelluloses or hydroxypropyl methylcellulose or dextrin or wheat starch).tw. (5004)
33 Inulin.tw. or Inulin/ (11472)
34 (sterculia or Konjac mannan).tw. (203)
35 (Legume or bean fiber or Lignin or Locust bean or Methylcellulose).tw. (27150)
36 Oligofructose.tw. (509)
37 (gum adj2 (Okra or guar or arabic or karaya or carob or tara or xanthan)).tw. (3946)
38 (pea hull or Pectin or Polydextrose or Psyllium or Ispaghula).tw. (9069)
39 Chemically modified starch.tw. (20)
40 (high amylose starch or high amylase starch).tw. (154)
41 (Xylans or Xyloglucans).tw. (815)
42 (Benefiber or Citrucel or FiberChoice or Fibersure or Hi-Maize or Konsyl or Fibersol or Metamucil or Normacol).tw. (116)
43 (Actilight or Meiologio or NutraFlora or neosugar or Normafib or Orafti or Synergy1).tw. (69)
44 (Litesse or Nutriose or Novelose or Fibersym).tw. (58)
45 (actistar or BranaVita or Fibrulose or GrainWise or Oliggo-Fiber or Oliggofiber or Fibruline or naturaflora or normafibe or beneo synergy or novelose).tw. (23)
46 or/22-45 (189103)
47 micronutrient*.tw. or exp Micronutrients/ (718342)
48 (vitamin* or provitamins or "vitamin B complex").tw. or exp Vitamins/ (416707)
49 (retinol or "retinyl esters" or "provitamin A carotenoid*" or "vitamin A").tw. (32743)
50 (thiamin or thiamine or "vitamin B1" or "vitamin B 1").tw. (13861)
51 (riboflavin or "vitamin B2" or "vitamin B 2").tw. (10821)
52 (niacin or "vitamin B3" or "vitamin B 3").tw. (5005)
53 ("pantothenic acid" or "vitamin B5" or "vitamin B 5").tw. (1913)
54 (pyridoxal or pyridoxamine or pyridoxine or "vitamin B6" or "vitamin B 6").tw. (18007)
55 (biotin or "vitamin B7" or "vitamin B 7").tw. (27323)
56 (folate or "folic acid" or "vitamin B9" or "vitamin B 9").tw. (40898)
57 (cobalamin or "vitamin B12" or "vitamin B 12").tw. (23375)
58 ("ascorbic acid" or "vitamin C").tw. (47781)
59 (calciferol* or cholecalciferol* or ergocalciferol* or "vitamin D" or calcidiol or "25(OH)D").tw. (65088)
60 (alpha-tocopherol* or a-tocopherol or tocopherol* or tocotrienol* or "vitamin E").tw. (41940)
61 (phylloquinone or phytomenadione or phytonadione or "vitamin K" or "vitamin K1" or "vitamin K 1").tw. (16268)
62 (choline or "vitamin bp" or "vitamin b p").tw. (38205)
63 (mineral* or macromineral* or micromineral* or "trace element*").tw. or exp Trace Elements/ (529764)
64 calcium.tw. (366395)
65 chloride*.tw. (129764)
66 chromium.tw. (22089)

67 copper.tw. (84523)
68 (iodine or iodide).tw. (70563)
69 iron.tw. (172733)
70 fluoride*.tw. (38392)
71 magnesium.tw. (53642)
72 manganese.tw. (28834)
73 molybdenum.tw. (7471)
74 (phosphorus or phosphorous or phosphate*).tw. (302458)
75 potassium.tw. (128900)
76 selenium.tw. (27433)
77 sodium.tw. (332810)
78 sulfur.tw. (43620)
79 zinc.tw. (107578)
80 or/47-79 (2201714)
81 exp Nutritional Requirements/ (22082)
82 ((nutrition* or dietary) adj requirement*).tw. (4176)
83 Recommended Dietary Allowances/ (2181)
84 ((recommended or reference) adj (daily or dietary or nutrient) adj (intake* or allowance*)).tw. (3847)
85 ((dietary reference adj (intake* or value*)) or (DRI or DRIs or DRV or DRVs)).tw. (2942)
86 ("adequate intake" or "average requirement" or "reference intake" or "tolerable upper intake level" or "chronic disease risk reduction").tw. (2380)
87 or/81-86 (31160)
88 80 and 87 (14243)
89 46 or 80 or 88 (2351732)
90 21 and 89 (51640)
91 ((diet* or eating or food* or nutrient* or nutrition) adj5 (adequa* or adhere* or allowance* or compliance or comply* or composition or diversity or guide* or habit* or health* or ideal or intake* or preference* or prudent or quality or recommend* or require* or risk* or unhealth* or variety) adj5 (assess* or indicator* or index* or indices or measur* or metric* or pattern* or profile* or questionnaire* or reference* or scale* or score* or scoring or tool*)).tw. (45768)
92 diet*.tw. or exp Diet/ (688924)
93 (assess* or indicator* or index* or indices or measur* or metric* or pattern* or profile* or questionnaire* or reference* or scale* or score* or scoring or tool*).tw. (8744481)
94 (quality or variety or diversity).tw. (1671101)
95 92 and 93 and 94 (36081)
96 nutrition assess*.tw. or exp Nutrition Assessment/ (17664)
97 94 and 96 (2217)
98 (AHEI or CCDI or CHEI or DAS or DDI or DDP or DDS or FAVVA or FVI or (fruit and vegetable intake) or FVS or HDI or HEI or HFI or HLD-Index or HPVS or KDAGCAI or KidMed or N-DDP or NIS or NutricheQ or RFS or TFS).tw. (65792)
99 (FCS and consum*).tw. (88)
100 ((MDP or MDS or MDQI or MEDAS or MEDLIFE) and Mediterranean).tw. (438)
101 ((NRF and rich) or Nutrient Rich Food*).tw. (312)
102 or/98-101 (66527)
103 (diet* or eating or food* or nutrient* or nutrition).tw. (1174282)

104 93 and 102 and 103 (5532)
105 (Baltic Sea Diet Score or Chinese Children Dietary Index or The Diet Score or DGA Adherence Index or Expected Food Pattern or Food Consumption Score or Food Index or (Fruit and Vegetable Variety Score) or Healthy Lifestyle Index or Healthy Preference Index or Korean Dietary Action Guides for Children Adherence Index or (Infant and Child Feeding Index) or MDD-W or Dietary Diversity for Women or Mean Adequacy Ratio* or Mediterranean Diet Adherence Screener or Norwegian Adolescent Diet Score or Nutrient Adequacy Ratio* or Variety Index for Toddlers or Vegetarian Lifestyle Index).tw. (1627)
106 (("Mediterranean" or "Dietary Approaches to Stop Hypertension" or DASH) adj3 (assess* or indicator* or index* or indices or measur* or metric* or pattern* or profile* or questionnaire* or reference* or scale* or score* or scoring or tool*)).tw. (6065)
107 91 or 95 or 97 or 104 or 105 or 106 (80926)
108 21 and 107 (4108)
109 90 or 108 (54294)
110 limit 109 to (english language and humans) (15401)
111 limit 110 to "all infant (birth to 23 months)" (963)
112 ((address or autobiography or bibliography or biography or comment or congress or dataset or dictionary or directory or editorial or electronic supplementary materials or "expression of concern" or guideline or interactive tutorial or interview or lecture or legal case or legislation or letter or meta analysis or news or newspaper article or observational study, veterinary or patient education handout or periodical index or personal narrative or portrait or practice guideline or randomized controlled trial, veterinary or "review" or technical report or video-audio media or webcast) not (Clinical Trial or Controlled Clinical Trial or Randomized Controlled Trial or Observational Study or Comparative Study)).pt. (5225894)
113 110 not (111 or 112) (12669)
114 limit 113 to yr="2010 -Current" (6214)

****

Table S1. Publications excluded from the review and reasons for exclusion.

No.	Author	Year	Title	Exclusion reason
1	Aggarwal and Drewnowski	2019	Plant- and animal-protein diets in relation to sociodemographic drivers, quality, and cost: findings from the Seattle Obesity Study	no sugar/diet quality association
2	An	2016	Beverage Consumption in Relation to Discretionary Food Intake and Diet Quality among US Adults, 2003 to 2012	sugar not quantified
3	Barr	2018	Breakfast in Canada: Prevalence of Consumption, Contribution to Nutrient and Food Group Intakes, and Variability across Tertiles of Daily Diet Quality. A Study from the International Breakfast Research Initiative	sugar not quantified in total diet
4	Barraj	2019	Comparisons of Nutrient Intakes and Diet Quality among Water-Based Beverage Consumers	no sugar/diet quality association
5	Batal	2018	Quantifying associations of the dietary share of ultra-processed foods with overall diet quality in First Nations peoples in the Canadian provinces of British Columbia, Alberta, Manitoba and Ontario	no diet quality outcome
6	Bell	2019	Dietary patterns and risk of obesity and early childhood caries in Australian toddlers: findings from an Australian cohort study	wrong population
7	Bilici	2018	Assessment of the Contribution of Dietary and Beverage Intake Quality to Obesity Development	sugar not quantified
8	Blumfield	2021	Balanced carbohydrate ratios are associated with improved diet quality in Australia: A nationally representative cross-sectional study	no diet quality outcome
9	Braz	2018	Added sugar intake by adolescents: A population-based study	sugar not quantified
10	Brown	2022	Healthy plant-based diets and their short-term effects on weight loss, nutrient intake and serum cholesterol levels	no diet quality outcome
11	Byker Shanks	2022	The UnProcessed Pantry Project (UP3): A Community-Based Intervention Aimed to Reduce Ultra-Processed Food Intake Among Food Pantry Clients	no sugar/diet quality association
12	Calixto Andrade	2021	Consumption of Ultra-Processed Food and Its Association with Sociodemographic Characteristics and Diet Quality in a Representative Sample of French Adults	no sugar/diet quality association
13	Caspi	2021	Applying the Healthy Eating Index-2015 in a Sample of Choice-Based Minnesota Food Pantries to Test Associations Between Food Pantry Inventory, Client Food Selection, and Client Diet	no sugar/diet quality association

14	Colomier	2022	A BETTER DIET QUALITY IS ASSOCIATED WITH MILDER SYMPTOM PATTERNS IN PATIENTS WITH IRRITABLE BOWEL SYNDROME (IBS)	wrong publication type
15	Colucci	2011	Effect of added sugars on nutrient intake among adolescents living in the city of São Paulo, Brazil	wrong publication type
16	de Moraes	2021	An Ultra-Processed Food Dietary Pattern Is Associated with Lower Diet Quality in Portuguese Adults and the Elderly: The UPPER Project	no diet quality outcome
17	Dello Russo	2018	The Impact of Adding Sugars to Milk and Fruit on Adiposity and Diet Quality in Children: A Cross-Sectional and Longitudinal Analysis of the Identification and Prevention of Dietary- and Lifestyle-Induced Health Effects in Children and Infants (IDEFICS) Study	sugar not quantified
18	Doherty	2021	Sugar-sweetened beverage (SSB) consumption is associated with lower quality of the non-SSB diet in US adolescents and young adults	sugar not quantified
19	Doostan	2016	Assessment of the dietary pattern of dormitory students in Kerman, Iran	sugar not quantified
20	Drewnowski	2018	Breakfast in the United States: Food and Nutrient Intakes in Relation to Diet Quality in National Health and Examination Survey 2011-2014. A Study from the International Breakfast Research Initiative	sugar not quantified
21	Elorriaga	2017	Is drinking mate associated with poor diet quality and food and nutrient intakes among South American adults?	wrong publication type
22	Escobedo Avila	2022	Evaluation of Common Bean Baked Snack Consumption on Subjective Satiety, Energy Intake and Glycemic Response in People With Overweight and Normal Weight	wrong publication type
23	Fagt	2018	Breakfast in Denmark. Prevalence of Consumption, Intake of Foods, Nutrients and Dietary Quality. A Study from the International Breakfast Research Initiative	sugar not quantified in total diet
24	Fiorito	2010	Girls' early sweetened carbonated beverage intake predicts different patterns of beverage and nutrient intake across childhood and adolescence	no sugar/diet quality association
25	Fontes	2019	Increased sugar-sweetened beverage consumption is associated with poorer dietary quality: a cross-sectional population-based study	sugar not quantified
26	Froberg	2022	Leisure-time organised physical activity and dietary intake among Swedish adolescents	no diet quality outcome

27	Gaal	2018	Breakfast in relation to overall diet quality in UK children and adolescents	wrong publication type
28	Gaal	2018	Breakfast Consumption in the UK: Patterns, Nutrient Intake and Diet Quality. A Study from the International Breakfast Research Initiative Group	sugar not quantified in total diet
29	Gamba	2019	Sugar sweetened beverage consumption during pregnancy is associated with lower diet quality and greater total energy intake	sugar not quantified
30	Garcia-Guerra	2022	Consumption of Micronutrient Powder, Syrup or Fortified Food Significantly Improves Zinc and Iron Status in Young Mexican Children: A Cluster Randomized Trial	no sugar intervention
31	Halliday	2018	Adolescents perceive a low added sugar adequate fiber diet to be more satiating and equally palatable compared to a high added sugar low fiber diet in a randomized-crossover design controlled feeding pilot trial	no diet quality outcome
32	Harvie	2017	Long-term irritable bowel syndrome symptom control with reintroduction of selected FODMAPs	no sugar intervention
33	Hedrick	2015	Changes in the Healthy Beverage Index in Response to an Intervention Targeting a Reduction in Sugar-Sweetened Beverage Consumption as Compared to an Intervention Targeting Improvements in Physical Activity: Results from the Talking Health Trial	sugar not quantified
34	Hedrick	2017	Dietary quality changes in response to a sugar-sweetened beverage-reduction intervention: results from the Talking Health randomized controlled clinical trial	no sugar/diet quality association
35	Higashiguchi	2010	Calcium intake and associated factors in a general Japanese population: baseline data of NIPPON DATA80/90 and the National Nutrition Survey	sugar not quantified in total diet
36	Huth	2013	Major food sources of calories, added sugars, and saturated fat and their contribution to essential nutrient intakes in the U.S. diet: data from the National Health and Nutrition Examination Survey (2003-2006)	no sugar intervention
37	Kachurak	2019	Daily Snacking Occasions, Snack Size, and Snack Energy Density as Predictors of Diet Quality among US Children Aged 2 to 5 Years	no sugar/diet quality association
38	Keast	2011	Dried fruit consumption is associated with improved diet quality and reduced obesity in US adults: National Health and Nutrition Examination Survey, 1999-2004	sugar not quantified

39	Larson	2011	Young adults and eating away from home: associations with dietary intake patterns and weight status differ by choice of restaurant	sugar not quantified
40	Lauria	2021	Ultra-processed foods consumption and diet quality of European children, adolescents and adults: Results from the I.Family study	no sugar/diet quality association
41	Lee	2019	Diet Quality and Fruit, Vegetable, and Sugar-Sweetened Beverage Consumption by Household Food Insecurity among 8- to 12-Year-Old Children during Summer Months	no sugar intervention
42	Leung	2018	Sugar-Sweetened Beverage and Water Intake in Relation to Diet Quality in U.S. Children	sugar not quantified
43	Liu	2022	Consumption of Ultraprocessed Foods and Diet Quality Among U.S. Children and Adults	sugar not quantified
44	Liu	2022	Evaluation of Dietary Quality Based on Intelligent Ordering System and Chinese Healthy Eating Index in College Students from a Medical School in Shanghai, China	sugar not quantified
45	Loth	2020	The Contribution of Snacking to Overall Diet Intake among an Ethnically and Racially Diverse Population of Boys and Girls	sugar not quantified in total diet
46	Lowndes	2011	High-fructose corn syrup and sucrose are nutritionally equivalent and may help improve dietary quality during weight loss	wrong publication type
47	Lyons	2015	Food portion sizes and dietary quality in Irish children and adolescents	sugar not quantified
48	Lyu	2022	The Effect of a Multifaceted Intervention on Dietary Quality in Schoolchildren and the Mediating Effect of Dietary Quality between Intervention and Changes in Adiposity Indicators: A Cluster Randomized Controlled Trial	sugar not quantified
49	Maillot	2018	Beverage consumption patterns among 4-19 y old children in 2009-14 NHANES show that the milk and 100% juice pattern is associated with better diets	no sugar/diet quality association
50	Maldonado-Pereira	2022	Evaluation of the nutritional quality of ultra-processed foods (ready to eat + fast food): Fatty acids, sugar, and sodium	no sugar intervention
51	Marriott	2010	Intake of added sugars and selected	no sugar/diet quality association
52	Mastrangelo	2022	Association between the consumption of sugar-sweetened beverages and food markers: National Dietary Survey 2008-2009	sugar not quantified
53	Mousavi	2021	Adherence to Alternative Healthy Eating Index (AHEI-2010) is not associated with risk of stroke in Iranian adults: A case-control study	sugar not quantified

54	Mullie	2016	Relation Between Sugar-Sweetened Beverage Consumption, Nutrition, and Lifestyle in a Military Population	sugar not quantified
55	Mullie	2018	Relation between sugar-sweetened beverage consumption and micronutrient intake in a prospective study	sugar not quantified
56	Murakami	2018	Breakfast in Japan: Findings from the 2012 National Health and Nutrition Survey	sugar not quantified in total diet
57	Murakami	2022	Characterisation of breakfast, lunch, dinner and snacks in the Japanese context: an exploratory cross-sectional analysis	sugar not quantified in total diet
58	Murakami and Livingstone	2016	Associations between meal and snack frequency and diet quality and adiposity measures in British adults: findings from the National Diet and Nutrition Survey	no sugar/diet quality association
59	Narayana	2021	Low Fodmap Diet Adherence Is High and Positively Impacts Nutritional Intake and Diet Quality in Children with Irritable Bowel Syndrome (Ibs)	wrong publication type
60	Narayana	2022	Children with functional abdominal pain disorders successfully decrease FODMAP food intake on a low FODMAP diet with modest improvements in nutritional intake and diet quality	no sugar/diet quality association
61	Okubo	2016	Early sugar-sweetened beverage consumption frequency is associated with poor quality of later food and nutrient intake patterns among Japanese young children: the Osaka maternal and child health study	sugar not quantified
62	Olza	2019	Adequacy of Critical Nutrients Affecting the Quality of the Spanish Diet in the ANIBES Study	no sugar/diet quality association
63	O'Neil	2010	Relationship between 100% juice consumption and nutrient intake and weight of adolescents	sugar not quantified
64	O'Neil	2011	Candy consumption was not associated with body weight measures, risk factors for cardiovascular disease, or metabolic syndrome in US adults: NHANES 1999-2004	no sugar/diet quality association
65	O'Neil	2011	Diet quality is positively associated with 100% fruit juice consumption in children and adults in the United States: NHANES 2003-2006	no sugar/diet quality association
66	O'Neil	2011	Association of candy consumption with body weight measures, other health risk factors for cardiovascular disease, and diet quality in US children and adolescents: NHANES 1999-2004	no sugar/diet quality association

67	O'Neil	2012	Presweetened and nonpresweetened ready-to-eat cereals at breakfast are associated with improved nutrient intake but not with increased body weight of children and adolescents: NHANES 1999-2002	no sugar/diet quality association
68	O'Neil	2014	Nutrient intake, diet quality, and weight/adiposity parameters in breakfast patterns compared with no breakfast in adults: National Health and Nutrition Examination Survey 2001-2008	sugar not quantified
69	Parker	2022	Preliminary Assessment of the Healthy Beverage Index for US Children and Adolescents: A Tool to Quantify the Overall Beverage Intake Quality of 2- to 19-Year Olds	sugar not quantified
70	Pereira	2017	Proposal for a breakfast quality index for brazilian population: Rationale and application in the Brazilian National Dietary Survey	no diet quality outcome
71	Piernas	2014	Low-calorie- and calorie-sweetened beverages: diet quality, food intake, and purchase patterns of US household consumers	sugar not quantified
72	Pinto	2019	Assessment of Diet Quality in Chilean Urban Population through the Alternate Healthy Eating Index 2010: A Cross-Sectional Study	sugar not quantified
73	Poston	2022	Adaptation of a Modified Diet Quality Index to Quantify Healthfulness of Food-Related Toy Sets	sugar not quantified
74	Reister and Leidy	2020	An afternoon hummus snack affects diet quality, appetite, and glycemic control in healthy adults	sugar not quantified
75	Robert	2022	Associations between Resilience and Food Intake Are Mediated by Emotional Eating in the NutriNet-Sante Study	sugar not quantified
76	Romo-Palafox	2018	Contribution of Beverage Selection to the Dietary Quality of the Packed Lunches Eaten by Preschool-Aged Children	wrong population
77	Roth	2022	Poor intake of vitamins and minerals is associated with symptoms among patients with irritable bowel syndrome	sugar not quantified
78	Ruiz	2018	Breakfast Consumption in Spain: Patterns, Nutrient Intake and Quality. Findings from the ANIBES Study, a Study from the International Breakfast Research Initiative	sugar not quantified in total diet
79	Rusmevichientong	2018	The association between types of soda consumption and overall diet quality: evidence from National Health and Nutrition Examination Survey (NHANES)	sugar not quantified
80	Ryan	2022	Exploring the Intersection Between Diet and Self-Identity: A Cross-Sectional Study With Australian Adults	sugar not quantified

81	Santiago-Torres	2014	Home food availability, parental dietary intake, and familial eating habits influence the diet quality of urban Hispanic children	sugar not quantified
82	Schwedhelm	2021	Using food network analysis to understand meal patterns in pregnant women with high and low diet quality	sugar not quantified
83	Shan	2019	Trends in Dietary Carbohydrate, Protein, and Fat Intake and Diet Quality Among US Adults, 1999-2016	sugar not quantified
84	Shih	2019	Increased coffee, tea, or other sugar-sweetened beverage consumption in adolescents is associated with less satisfactory dietary quality, body fatness and serum uric acid profiles over the past 18 years in Taiwan	sugar not quantified
85	Shim	2022	Association between Ultra-processed Food Consumption and Dietary Intake and Diet Quality in Korean Adults	sugar not quantified
86	Smith	2021	Association between whole grain food intake in Canada and nutrient intake, food group intake and diet quality: findings from the 2015 Canadian Community Health Survey	no diet quality outcome
87	Spaniol	2021	Early consumption of ultra-processed foods among children under 2 years old in Brazil	no sugar/diet quality association
88	Staudacher	2020	Nutrient Intake, Diet Quality, and Diet Diversity in Irritable Bowel Syndrome and the Impact of the Low FODMAP Diet	no sugar/diet quality association
89	Steele	2017	The share of ultra-processed foods and the overall nutritional quality of diets in the US: evidence from a nationally representative cross-sectional study	no sugar/diet quality association
90	Tallman	2020	Dietary Patterns and Health Outcomes among African American Maintenance Hemodialysis Patients	wrong population
91	Tao	2022	Trends in Diet Quality by Race/Ethnicity among Adults in the United States for 2011-2018	no sugar/diet quality association
92	Thompson	2021	Earlier introduction to sugar-sweetened beverages associated with lower diet quality among WIC children at age 3 years	sugar not quantified
93	Thomson	2011	Food and beverage choices contributing to dietary guidelines adherence in the Lower Mississippi Delta	no sugar intervention
94	Thomson	2011	Simulated reductions in consumption of sugar-sweetened beverages improves diet quality in Lower Mississippi Delta adults	sugar not quantified
95	Tigchelaar	2017	Habitual diet and diet quality in Irritable Bowel Syndrome: A case-control study	no sugar/diet quality association
96	Tomiya	2022	Association between diet quality index, food and nutrient intake and metabolic parameters	sugar not quantified

			in adolescents from Recife, northeastern Brazil	
97	Tripicchio	2022	More-To-Less Home Healthful Foods Ratio is Associated With Diet Quality in Preadolescents With Elevated Body Mass Index	sugar not quantified
98	Turner-McGrievy	2021	Examining commonalities and differences in food groups, nutrients, and diet quality among popular diets	no diet quality outcome
99	Wang	2014	Trends in dietary quality among adults in the United States, 1999 through 2010	sugar not quantified
100	Wanselius	2019	Procedure to Estimate Added and Free Sugars in Food Items from the Swedish Food Composition Database Used in the National Dietary Survey Riksmaten Adolescents 2016-17	no diet quality outcome
101	Wedde	2020	Associations between Family Meal Context and Diet Quality among Preschool-Aged Children in the Guelph Family Health Study	sugar not quantified
102	Woo	2021	Longitudinal Diet Quality Trajectories Suggest Targets for Diet Improvement in Early Childhood	sugar not quantified

Table S2. Risk of bias assessment results for all included studies using an adapted NUQUEST Cohort Checklist for Nutrition Studies[1].

	**Domain: Selection**		**Domain: Comparability**		**Domain: Outcomes**	**Domain: Nutrition-specific**			**Domain ratings and overall ROB[2]**
**Study, year (ref)**	**1.1**	**1.4**	**1.7**	**1.8**	**1.9**	**2.1a**	**2.1b**	**2.2**	
Castellanos-Gutierrez et al., 2021 (1)	Yes	Yes	No	No	Yes	Yes	Yes	Yes	+-++<br>Poor
Chiavaroli et al., 2022 (2)	Yes	Yes	No	No	Yes	Yes	Yes	Yes	+-++<br>Poor
Fujiwara et al., 2020 (3)	Yes	Yes	No	Probably no	Yes	Yes	Yes	Yes	+-++<br>Poor
Fulgoni et al., 2019 (4)	Yes	Yes	Yes	No	Yes	Yes	Yes	Yes	+0++<br>Neutral
Fulgoni et al., 2020 (5)	Yes	Yes	Yes	No	Yes	Yes	Yes	Yes	+0++<br>Neutral
Gibson et al., 2016 (6)	Yes	Yes	Yes	No	Yes	Yes	Probably Yes	Yes	+0++<br>Neutral
Goletzke et al., 2015 (7)	Yes	Yes	Yes	Probably yes	Yes	Yes	Probably Yes	Yes	++++<br>Neutral
Golley et al., 2011 (8)	Yes	Yes	Yes	No	Yes	Yes	Probably Yes	Yes	+0++<br>Neutral
Gomez et al., 2020 (9)	Yes	Yes	Yes	No	Yes	Probably yes	Probably Yes	Yes	+0++<br>Neutral
González-Padilla et al., 2020 (10); Riksmaten Adults	Yes	Yes	Yes	Probably no	Yes	Yes	Probably Yes	Yes	+0++<br>Neutral
González-Padilla et al., 2020 (10); Malmö Diet and Cancer Study	Yes	Yes	Yes	Probably no	Yes	Yes	Probably Yes	Yes	+0++<br>Neutral
Gress et al., 2020 (11)	Yes	Yes	Yes	Probably yes	Yes	Yes	Yes	Yes	++++<br>Neutral
Kaartinen et al., 2017 (12)	Yes	Yes	No	Yes	Yes	Yes	Probably Yes	Yes	+0++<br>Neutral
MacIntyre et al., 2012 (13)	Yes	Yes	No	No	Yes	Yes	Probably Yes	Yes	+-++<br>Poor

Maunder et al., 2015 (14)	Yes	Yes	No	No	Yes	Yes	Yes	Yes	+-++<br>Poor
Meira et al., 2021 (15)	Yes	Yes	No	No	Yes	Probably yes	Probably Yes	Yes	+-++<br>Poor
Montejano Vallejo et al., 2022 (16)	Yes	Yes	Yes	No	Yes	Yes	Probably Yes	Yes	+0++<br>Neutral
Moraeus et al., 2020 (17)	Yes	Yes	Yes	No	Yes	Yes	Yes	Yes	+0++<br>Neutral
Morales-Suarez-Varela et al., 2020 (18)	Yes	Yes	Yes	Probably no	Yes	Yes	Yes	Yes	+0++<br>Neutral
Moshtaghian et al., 2016 (19)	Yes	Yes	No	Probably no	Yes	Yes	Yes	Yes	+-++<br>Poor
Murakami et al., 2018 (20)	Yes	Yes	Yes	Probably yes	Yes	Yes	Yes	Yes	++++<br>Neutral
Sluik et al., 2016 (21)	Yes	Yes	Yes	No	Yes	Yes	Probably Yes	Yes	+0++<br>Neutral
Voortman et al., 2015 (22)	Yes	Yes	No	No	Yes	Yes	Probably Yes	Yes	+-++<br>Poor
Wang et al., 2015 (23)	Yes	Yes	Yes	No	Yes	Yes	Yes	Yes	+0++<br>Neutral
Wang et al., 2020 (24)	Yes	Yes	No	Yes	Yes	Yes	Yes	Yes	+0++<br>Neutral
Wong et al., 2014 (25)	Yes	Yes	No	No	Yes	Yes	Probably Yes	Yes	+-++<br>Poor
Wong et al., 2019 (26)	Yes	Yes	Yes	Probably no	Yes	Yes	Probably Yes	Yes	+0++<br>Neutral

24 HR, 24 hour recall; FFQ, food frequency questionnaire; NUQUEST, NUtrition QUality Evaluation Strengthening Tool: Development of tools for the evaluation of risk of bias in nutrition studies (27); ROB, risk of bias

[1] The following NUQUEST items applicable to cross-sectional studies and two newly created items were used in this review and included in the study domain ratings:

1.1: The participants being studied are selected from source populations that are comparable in all respects other than the exposure under investigation (i.e., sugar intake).
1.4: The exposure assessment method is adequate to differentiate exposure among study participants (i.e., 24 HR, diet record, FFQ, interview with dietician).
1.7: Exposure level or prognostic factor is assessed more than once.
1.8: The main potential confounders are identified and taken into account in the design and/or analysis (i.e., age, sex, body mass index, a socioeconomic indicator such as income or education level, health status, if not all healthy); Yes (all confounders), Probably yes (4/5 confounders), Probably no (3/5 confounders), No (<3 confounders).
1.9: The outcomes of the study are clearly defined.
2.1: The frequency and quantity of the exposure under study are accurately and reliably measured. [This question was replaced with 2.1a and 2.1b below]
2.1a: The tool used to assess the exposure level was specific to the country or population included in this study (e.g., a national nutrient database)

2.1b: The tool used to assess the exposure level was appropriate for the specific exposure of interest in the study (e.g., the study used a database with data on added sugar)
2.2: The relevant exposure at baseline is measured and taken into account.

[2] Risk of bias ratings for the four NUQUEST domains and the overall study were assessed using the following criteria; After making initial judgements, all overall study ratings were downgraded one level due to the cross-sectional study design:

Good (+): Almost all criteria met with little or no concern over the study design and a low RoB.
Neutral (0): Most criteria met. There are some flaws in the study, with an associated concern over the study design. Moderate RoB
Poor (-): Most or all criteria not met, or significant flaws related to key aspects of the study design. High RoB.

Table S3. Results for all included studies reporting associations for sugar intake with diet quality measured by indexes or scores.

Study, year (ref)	Sugar type (unit)	Index or score; description (points possible)	Sugar component(s) included in the index or score	Subgroup	Association reported[1]	Analysis for measures of effect	Confounders accounted for in analysis; in design
Castellanos-Gutierrez et al., 2021 (1)	Added sugars (g)	Global Diet Quality Score (GDQS); 25 food groups scored according to their health implications (0-49)	SSBs, sweets, and ice cream (which include sugars and sweeteners, among other foods)	24HR	---	Spearman correlations for index scores and sugar intake	Total energy; sex (all women); age group (15–49 y)
				FFQ	---		
		Global Diet Quality Score positive submetric (GDQS+); 16 healthy food groups with higher scores assigned to higher intakes (0–32)	None	24HR	---		
				FFQ	---		
		Global Diet Quality Score negative submetric (GDQS–); 9 unhealthy food groups with higher scores assigned to lower intakes except that high fat dairy and red meat are assigned higher scores for moderate intake and lower scores for very low or very high intakes (0–17)	SSBs, sweets, and ice cream (which include sugars and sweeteners, among other foods)	24HR	---		
				FFQ	---		
		Alternate Healthy Eating Index-2010 (AHEI-2010); 10 foods and nutrients predictive of chronic disease risk, excluding the alcohol component (0–100)	SSBs and fruit juice	24HR	---		
				FFQ	---		
		Minimum Dietary Diversity for Women (MDD-W); FAO indicator for 10 recommended food groups with points assigned for intake ≥15 g/d (0-10)	None	24HR	---		
				FFQ	---		
Golley et al., 2011 (8)	Total sugars (g)	Dietary Guideline Index for Children and Adolescents (DGI-CA); 11 food-based components related to the Dietary Guidelines for Children and Adolescents in Australia (0–100)	None		---	Complex samples: general linear model for sugar intake across quintiles of index scores	Age; age group (4–16 y)

Study, year (ref)	Sugar type (unit)	Index or score; description (points possible)	Sugar component(s) included in the index or score	Subgroup	Association reported[1]	Analysis for measures of effect	Confounders accounted for in analysis; in design
Gomez et al., 2020 (9)	Added sugars (g/1,000 kcal)	Minimum Dietary Diversity for Women (MDD-W) Dietary Diversity Score (DDS); FAO indicator for 10 recommended food groups with points assigned for intake ≥15 g/d (0–10)	None		---	Presumably, Student's t-test on sugar intake for DDS scores <5 (low diversity) v ≥5 (high diversity)	None; sex (all women); age group (15–49 y); nutrient density
Maunder et al., 2015 (14)	Added sugars (%E)	Dietary Diversity Score (DDS); count of dietary intake from 9 food groups (0–9)	None		+ (Q1 v Q2)<br>+ (Q1 v Q3)<br>+ (Q1 v Q4)<br>0 (Q2 v Q3)<br>+ (Q2 v Q4)<br>0 (Q3 v Q4)	Bonferroni comparisons for index scores between quartiles of sugar intake	None; age group (4–8 y); nutrient density
		Food Variety Score (FVS); total number of different food items consumed in 24 hours (range NR)	None		+ (Q1 v Q2)<br>+ (Q1 v Q3)<br>+ (Q1 v Q4)<br>0 (Q2 v Q3)<br>+ (Q2 v Q4)<br>0 (Q3 v Q4)		
		Mean Adequacy Ratio (MAR)[3]; mean of the nutrient adequacy ratios for 11 micronutrients based on recommended nutrient intakes (RNIs) (0–100)	None		+ (Q1 v Q2)<br>+ (Q1 v Q3)<br>0 (Q1 v Q4)<br>0 (Q2 v Q3)<br>0 (Q2 v Q4)<br>0 (Q3 v Q4)		
	Added sugars (g)	Dietary Diversity Score (DDS)			+++	Pearson's correlations for index scores and sugar intake	None; age group (4–8 y)
		Food Variety Score (FVS)			+++		
		Mean Adequacy Ratio (MAR)[3]			+++		
	Added sugars (g)	Dietary Diversity Score (DDS)			+++	Pearson's partial correlations for index scores and sugar intake	Energy intake; age group (4–8 y)
		Food Variety Score (FVS)			+++		
		Mean Adequacy Ratio (MAR)[3]			0		
Montejano Vallejo et al., 2022 (16)	Added sugars (%E)	Dietary Index (DI) score (internally developed); 18 food-based components from the EAT Lancet Reference Diet	All sweeteners		--	Linear regression for sugar intake across tertiles of index scores	Age; age group (15–17.1 y); nutrient density

Study, year (ref)	Sugar type (unit)	Index or score; description (points possible)	Sugar component(s) included in the index or score	Subgroup	Association reported[1]	Analysis for measures of effect	Confounders accounted for in analysis; in design
		(ELR-diet) with points assigned if intake met recommendations (0-18)					
Moraeus et al., 2020 (17)	Added sugars (%E)	Swedish Healthy Eating Index for Adolescents 2015 (SHEIA15); 9 food and nutrient components scored as the ratio between actual and recommended intakes based on key advice from 'Find your way' and the 2012 Nordic Nutrition Recommendations (0–9)	Less sugar		--- (Low v Med.)[4]<br>--- (Low v High)[4]<br>--- (Med. v High)[4]	One-way ANOVA with Bonferroni corrections for sugar intake between three index score groups (low, med., high)	None; age group (all adolescents); nutrient density
		Riksmaten Adolescents Diet Diversity Score (RADDS); 17 food subgroups based on food group diversity in 'Find your way' with points assigned for intakes >5g (0–17)	None		--- (Low v Med.)[4]<br>--- (Low v High)[4]<br>0 (Med. v High)		
Murakami et al., 2018 (20)	Total sugars (%E)	British Food Standard Agency (FSA) nutrient profiling system score of meals and snacks (total diet); nutrient content of foods and beverages consumed with positive points (0 to +10) assigned for energy, SFA, total sugar, and sodium content, and negative points (0 to -5) assigned for fruits/vegetables/nuts, dietary fiber, and protein content (-15 to 40).	Total sugar	4–10 y	---[5]	Linear regression for index scores and sugar intake	Age; sex; social class; age group; all healthy; nutrient density
				11–18 y	---[5]		
	Non-milk extrinsic sugar (%E)			4–10 y	---[5]		
				11–18 y	---[5]		
Sluik et al., 2016 (21)	Added sugars (%E)	Dutch Healthy Diet-index (DHD-index); 10 components for physical activity, foods, and nutrients based on the 2006 Dutch Guidelines for a Healthy Diet from the Dutch Health Council (0–100)	Consumption occasions with foods and beverages that contain easily fermentable sugars	Males	0 (<10 v ≥10 %E)<br>0 (<20 v ≥20 %E)	Independent t-test for sugar intake in guidelines adherent ("<" %E from sugar)	None; age group (19–69 y); nutrient density; sex

Study, year (ref)	Sugar type (unit)	Index or score; description (points possible)	Sugar component(s) included in the index or score	Subgroup	Association reported[1]	Analysis for measures of effect	Confounders accounted for in analysis; in design
			and drinks that are high in food acids with respect to dental health			and non-adherent ("≥" %E from sugar) groups	
				Females	0 (<10 v ≥10 %E)<br>--- (<20 v ≥20 %E)		
	Free sugars (%E)			Males	0 (<5 v ≥5 %E)<br>0 (<10 v ≥10 %E)		
				Females	0 (<5 v ≥5 %E)<br>0 (<10 v ≥10 %E)		
Voortman et al., 2015 (22)	Mono- and disaccharides (g)	Diet Quality Score for Preschool Children (internally developed); 10 food groups scored as ratios of reported and recommended intakes based on national and international food-based guidelines for preschool children and scientific literature (0–100)	Candy and snacks, SSBs		--	Linear regression for diet scores and energy-adjusted sugar intake	Sex; energy adjusted sugar intake; age (all were 25 months old)
Wang et al., 2015 (23)	Added sugars (g)	Canadian Healthy Eating Index (HEI-C); 9 food and nutrient components with scoring for youths aged 9–13 y based on the 2009 Eating Well with Canada's Food Guide (0–100)	"Other foods" mainly comprise foods rich in added sugars or solid fats		--- (solid foods)<br>--- (liquid foods)	ANOVA for index scores across tertiles of sugar intake	None; age group (8–10 y)
					Solid foods:<br>- (T1 v T2)<br>- (T1 v T3)<br>- (T2 v T3)<br>Liquid foods:<br>- (T1 v T2)<br>- (T1 v T3)<br>0 (T2 v T3)	Post hoc multiple comparisons (Duncan and Dunnett's T3 for equal and unequal variances, respectively) for index scores between tertiles of sugar intake	
		Canada's Food Guide for "Good diet" (proportion of population with HEI-C score ≥80); see above			Solid foods:<br>--- (overall)<br>- (T1 v T2)<br>- (T1 v T3)<br>- (T2 v T3)	Chi-squared for proportion of population with HEI-C score ≥80 among tertiles of sugar intake	

Study, year (ref)	Sugar type (unit)	Index or score; description (points possible)	Sugar component(s) included in the index or score	Subgroup	Association reported[1]	Analysis for measures of effect	Confounders accounted for in analysis; in design
					Liquid foods:<br>-- (overall)<br>- (T1 v T2)<br>- (T1 v T3)<br>0 (T2 v T3)		
Wong et al., 2014 (25)	Total sugars (g/MJ)	Healthy Dietary Habits Score for Adolescents (HDHS-A); 17 items grouped into 5 clusters based on existing scientific evidence and the NZ Food and Nutrition Guidelines for Healthy Children and Young People (0–68)	"Sugar sources" cluster includes fruit juice or drinks, soft drinks or energy drinks, and confectionary items		0	Linear trend analysis comparing sugar intake across tertiles of diet scores	None; age group (15–18 y); nutrient density
	Sucrose (g/MJ)				-		

24HR, 24 hour recall; %E, percent of total energy intake; ANOVA, analysis of variance; FFQ, food frequency questionnaire; g, grams; NR, not reported; Q, quantile; SSBs, sugar-sweetened beverages; T, tertile; v, versus; y, years

[1] Associations reported as: significant negative association (-, $P \leq 0.05$; --, $P \leq 0.01$; ---, $P \leq 0.001$); no association (0, $P > 0.05$); significant positive association (+, $P \leq 0.05$; ++, $P \leq 0.01$; +++, $P \leq 0.001$).
[2] Reported using non-milk extrinsic sugars as a proxy for free sugar.
[3] The Mean Adequacy Ratio (MAR) was calculated for 11 vitamins (A, B6, B12, C, thiamin, riboflavin, niacin, and folate) and minerals (calcium, iron, and zinc).
[4] Significance level not clearly reported, but $P \leq 0.001$ presumed based on all other reporting of statistical significance by the authors.
[5] Lower FSA scores reflect better nutritional quality. For consistency, this table presents the inverse association (e.g., higher sugar intake was associated with lower diet quality).

Table S4. Results for all included studies reporting associations for sugar intake with nutrients of public health concern (fiber, vitamin D, calcium, and potassium) and with sodium as a nutrient to limit.

Study, year (ref)	Sugar type (unit)	Nutrient (unit)	Subgroup	Association reported[1]	Analysis for measures of effect	Confounders accounted for in analysis; in design
**Fiber**						
Chiavaroli et al., 2022 (2)	Total sugars (%E)	Total fiber (g/1,000 kcal)	2–8 y	0	General linear model for fiber intake across quintiles of sugar intake	Misreporting status; sex; immigrant status; weekend reference day; age group; nutrient density
			9–13 y	0		
			14–18 y	+		
Fujiwara et al., 2020 (3)	Free sugars (%E)	Dietary fiber (g)		0	Linear regression for fiber intake across four sugar intake levels	Age; sex; weight status; age group (1–19 y); nutrient density
				+++ (<2.5 v 2.5 to <5 %E)<br>+++ (<2.5 v 5 to <10 %E)<br>0 (<2.5 v ≥10 %E)<br>0 (2.5 to <5 v 5 to <10 %E)<br>--- (2.5 to <5 v ≥10 %E)<br>0 (5 to <10 v ≥10 %E)	Tukey-Kramer test for fiber intake between four sugar intake levels (<2.5 %E, 2.5 to <5 %E, 5 to <10 %E, ≥10 %E)	
		Dietary fiber (g/1,000 kcal)		---	Linear regression for fiber intake across four sugar intake levels	
				0 (<2.5 v 2.5 to <5 %E)<br>0 (<2.5 v 5 to <10 %E)<br>--- (<2.5 v ≥10 %E)<br>--- (2.5 to <5 v 5 to <10 %E)<br>--- (2.5 to <5 v ≥10 %E)<br>--- (5 to <10 v ≥10 %E)	Tukey-Kramer test for fiber intake between four sugar intake levels (<2.5 %E, 2.5 to <5 %E, 5 to <10 %E, ≥10 %E)	
Fulgoni et al., 2020 (5)	Added sugars (%E)	Dietary fiber (percentage of the population above the AI)	2–18 y	Sugar intake decile was negatively associated with the percentage of the population above the AI for fiber, regardless of the method used (*P*-range: 0.0019 to < 0.0001)	Regression for percentage of population above the AI for fiber across deciles of sugar intake by various methods (1 day intake, 2 day average intake, UI/NCI, and UI/MCMC)	None; age group; nutrient density
			≥19 y	Sugar intake decile was negatively associated with the percentage of the population above the AI for fiber, regardless of the		

Study, year (ref)	Sugar type (unit)	Nutrient (unit)	Subgroup	Association reported[1]	Analysis for measures of effect	Confounders accounted for in analysis; in design
				method used (*P*-range: 0.0007 to 0.0002)		
Gibson et al., 2016 (6)	Free sugars (%E)[2]	Non-starch polysaccharide fiber (g/1,000 kcal)	4–10 y	---	ANOVA for fiber intake across quintiles of sugar intake	None; age group; nutrient density
				0 (all pairwise comparisons)	Bonferroni comparisons for fiber intake between quintiles of sugar intake	
			11–18 y	---	ANOVA for fiber intake across quintiles of sugar intake	
				0 (all pairwise comparisons)	Bonferroni comparisons for fiber intake between quintiles of sugar intake	
		Non-starch polysaccharide fiber (g)	4–10 y	0	ANOVA for fiber intake across quintiles of sugar intake	None; age group
			11–18 y	---		
				- (Q1 v Q5)<br>- (Q2 v Q5)<br>- (Q3 v Q5)<br>0 (all other comparisons)	Bonferroni comparisons for fiber intake between quintiles of sugar intake	
Goletzke et al., 2015 (7)	Total sugars (g/1,000 kJ)	Dietary fiber (g)		0	Cross-sectional estimate: Linear mixed regression model for increase/decrease in fiber intake at baseline associated with a 1 g/1,000 kJ increase in sugar intake	Age at study entry; prepregnancy BMI; ethnicity; intervention group; energy; sex (all female); all healthy; nutrient density
				0	20 wk concurrent change estimate: Linear mixed regression model for change in fiber intake associated with a 1 g/1,000 kJ increase in sugar intake over a mean 20 wk of gestation	
González-Padilla et al., 2020 (10)	Added sugars (%E)	Fiber (%E)	Riksmaten Adults	---	General linear model for fiber intake across six groups of sugar intake (<5 %E, 5 to 7.5 %E, 7.5 to 10 %E, 10 to 15 %E, 15 to 20 %E, >20 %E)	Age; sex; BMI; nonalcoholic energy intake; nutrient density
			Malmö Diet and Cancer Study	---		

Study, year (ref)	Sugar type (unit)	Nutrient (unit)	Subgroup	Association reported[1]	Analysis for measures of effect	Confounders accounted for in analysis; in design
Kaartinen et al., 2017 (12)	Added sucrose and fructose (g)	Dietary fiber (g)	Males	---	General linear model for fiber intake across quartiles of sugar intake	Age; energy intake; leisure-time physical activity; smoking status; education; BMI; sex
			Females	---		
	Naturally occurring sucrose and fructose (g)		Males	+++		
			Females	+++		
MacIntyre et al., 2012 (13)	Added sugars (g)	Fiber (g)	Males	+ (Q1 v Q4)<br>0 (all other comparisons)	Multivariate nonparametric tests for fiber intake between quartiles of added sugar intake	None; sex; all healthy
			Females	0 (Q1 v Q2)<br>+ (Q1 v Q3)<br>+ (Q1 v Q4)<br>+ (Q2 v Q3)<br>+ (Q2 v Q4)<br>0 (Q3 v Q4)		
Maunder et al., 2015 (14)	Added sugars (g)	Total fiber (unit NR)		+	Pearson's correlations for fiber intake and sugar intake	None; age group (4–8 y)
				---	Pearson's partial correlations for fiber intake and sugar intake	Energy intake; age group (4–8 y)
Meira et al., 2021 (15)	Free sugars (%E)	Dietary fiber (g/1,000 kcal)		---	Linear regression for sugar intake across quartiles of fiber intake with *F* test to determine significance of model	None; age group (10–19 y); nutrient density
Morales-Suarez-Varela et al., 2020 (18)	Simple sugars (g)	Fiber (g)	Males	+++	ANOVA for fiber intake across three groups of sugar intake (<25th percentile, 25th–75th percentile, >75th percentile)	None; age group (6–8 y); sex; all healthy
			Females	+++		
	Total sugars (%E)	Fiber (g)	Males	+++[3], non-linear		None; age group (6–8 y); sex; all healthy; nutrient density
			Females	++[3], non-linear		
Moshtaghian et al., 2016 (19)	Added sugars (%E)	Fiber (g)		---	Linear regression for fiber intake across three sugar intake categories	Age; sex; energy; BMI; nutrient density

Study, year (ref)	Sugar type (unit)	Nutrient (unit)	Subgroup	Association reported[1]	Analysis for measures of effect	Confounders accounted for in analysis; in design
				- (<5 v 5 to 10 %E)<br>- (<5 v >10 %E)<br>- (5 to 10 v >10 %E)	ANCOVA for fiber intake between three sugar intake categories	
Sluik et al., 2016 (21)	Added sugars (%E)	Fiber (component score, 0-10)	Males	--- (<10 v ≥10 %E)<br>--- (<20 v ≥20 %E)	Independent t-test for fiber intake between guidelines adherent ("<" %E from sugar) and non-adherent ("≥" %E from sugar) groups	None; age group (19–69 y); sex; nutrient density
			Females	--- (<10 v ≥10 %E)<br>--- (<20 v ≥20 %E)		
	Free sugars (%E)		Males	-- (<5 v ≥5 %E)<br>--- (<10 v ≥10 %E)		
			Females	--- (<5 v ≥5 %E)<br>--- (<10 v ≥10 %E)		
Wang et al., 2020 (24)	Total sugars (%E)	Total fiber (g/1,000 kcal)		+++	General linear model for fiber intake across quintiles of sugar intake	Age; sex; smoking; self-perceived health; blood pressure; diabetes; heart disease; cancer; osteoporosis; education; physical activity; income; BMI; immigrant status; weekend reference day; misreporting status; nutrient density
				+ (Q1 v Q3)<br>+ (Q1 v Q5)<br>0 (Q3 v Q5)	Post hoc comparisons with Bonferroni adjustments for fiber intake between quintiles of sugar intake (Q2 and Q4 NR)	
			Males (19–30 y)	0	General linear model for fiber intake across quintiles of total sugar intake	Age; sex; smoking; self-perceived health; blood pressure; diabetes; heart disease; cancer; osteoporosis; education; physical activity; income; BMI; immigrant status; weekend reference day; misreporting status; age group; sex; nutrient density
			Males (31–50 y)	+		
				0 (Q1 v Q3)<br>0 (Q1 v Q5)<br>0 (Q3 v Q5)	Post hoc comparisons with Bonferroni adjustments for fiber intake between quintiles of sugar intake (Q2 and Q4 NR)	
			Males (51–70 y)	+++	General linear model for fiber intake across quintiles of total sugar intake	

Study, year (ref)	Sugar type (unit)	Nutrient (unit)	Subgroup	Association reported[1]	Analysis for measures of effect	Confounders accounted for in analysis; in design
				0 (Q1 v Q3)<br>+ (Q1 v Q5)<br>0 (Q3 v Q5)	Post hoc comparisons with Bonferroni adjustments for fiber intake between quintiles of sugar intake (Q2 and Q4 NR)	
			Males (71+ y)	0	General linear model for fiber intake across quintiles of total sugar intake	
			Females (19–30 y)	0	General linear model for fiber intake across quintiles of total sugar intake	
			Females (31–50 y)	0		
			Females (51–70 y)	+++		
				0 (Q1 v Q3)<br>0 (Q1 v Q5)<br>0 (Q3 v Q5)	Post hoc comparisons with Bonferroni adjustments for fiber intake between quintiles of sugar intake (Q2 and Q4 NR)	
			Females (71+ y)	+	General linear model for fiber intake across quintiles of total sugar intake	
				0 (Q1 v Q3)<br>+ (Q1 v Q5)<br>0 (Q3 v Q5)	Post hoc comparisons with Bonferroni adjustments for fiber intake between quintiles of sugar intake (Q2 and Q4 NR)	
Wong et al., 2019 (26)	Free sugars (%E)	Dietary fiber (g)		---	Linear regression for fiber intake across six sugar cut-off levels (<5 %E, 5 to <10 %E, 10 to <15 %E, 15 to <20 %E, 20 to 25 %E, >25 %E)	Age; sex; socio-economic indices of disadvantage for areas (SEIFA); equivalized household income; remoteness of living area; age group (2–18 y); nutrient density
		Dietary fiber (g/1,000 kJ)		---		
**Vitamin D**						
Chiavaroli et al., 2022 (2)	Total sugars (%E)	Vitamin D (µg/1,000 kcal)	2–8 y	0	General linear model for vitamin D intake across quintiles of sugar intake	Misreporting status; sex; immigrant status; weekend reference day; age group; nutrient density
			9–13 y	0		
			14–18 y	0		
Fujiwara et al., 2020 (3)	Free sugars (%E)	Vitamin D (µg)		--	Linear regression for vitamin D intake across four sugar intake levels	Age; sex; weight status; age group (1–19 y); nutrient density
				0 (all pairwise comparisons)	Tukey-Kramer test for vitamin D intake between sugar intake levels (<2.5 %E, 2.5 to <5 %E, 5 to <10 %E, ≥10 %E)	

**Study, year (ref)**	**Sugar type (unit)**	**Nutrient (unit)**	**Subgroup**	**Association reported[1]**	**Analysis for measures of effect**	**Confounders accounted for in analysis; in design**
		Vitamin D (µg/1,000 kcal)		---	Linear regression for vitamin D intake across four sugar intake levels	
				--- (<2.5 v ≥10 %E)<br>0 (all other comparisons)	Tukey-Kramer test for vitamin D intake between sugar intake levels (<2.5 %E, 2.5 to <5 %E, 5 to <10 %E, ≥10 %E)	
Fulgoni et al., 2019 (4)	Added sugars (%E)	Vitamin D (percentage of population below the EAR)	2–8 y	Sugar intake decile was positively associated with the percentage of the population below the EAR for vitamin D (*P* = 0.007)	Linear regression for the percentage of the population below the EAR for vitamin D across deciles of sugar intake	Energy intake; age group; nutrient density
			9–18 y	Sugar intake decile was positively associated with the percentage of the population below the EAR for vitamin D (*P* = 0.0001)		
Fulgoni et al., 2020 (5)	Added sugars (%E)	Vitamin D (percentage of population below the EAR)	2–18 y	Sugar intake decile was positively associated with the percentage of the population below the EAR for vitamin D regardless of the method used (*P*-range: 0.04 to < 0.0001)	Regression for percentage of population below the EAR for vitamin D across deciles of sugar intake by various methods (1 day intake, 2 day average intake, UI/NCI, and UI/MCMC)	None; age group; nutrient density
			≥19 y	Sugar intake decile was not associated with the percentage of the population below the EAR for vitamin D for all methods (*P* > 0.06) except UI/MCMC which showed a positive association (*P* = 0.0001)		
Gibson et al., 2016 (6)	Free sugars (%E)[2]	Vitamin D (µg/4.18 MJ)	4–10 y	--	ANOVA for vitamin D intake across quintiles of sugar intake	None; age group; nutrient density
				0 (all pairwise comparisons)	Bonferroni comparisons for vitamin D intake between quintiles of sugar intake	
			11–18 y	---	ANOVA for vitamin D intake across quintiles of sugar intake	
				- (Q1 v Q5)<br>0 (all other comparisons)	Bonferroni comparisons for vitamin D intake between quintiles of sugar intake	

**Study, year (ref)**	**Sugar type (unit)**	**Nutrient (unit)**	**Subgroup**	**Association reported[1]**	**Analysis for measures of effect**	**Confounders accounted for in analysis; in design**
		Vitamin D (µg)	4–10 y	0	ANOVA for vitamin D intake across quintiles of sugar intake	None; age group
			11–18 y	-		
				0 (all pairwise comparisons)	Bonferroni comparisons for vitamin D intake between quintiles of sugar intake	
González-Padilla et al., 2020 (10)	Added sugars (%E)	Vitamin D (µg)	Riksmaten Adults	---	General linear model for vitamin D intake across six groups of sugar intake (<5 %E, 5 to 7.5 %E, 7.5 to 10 %E, 10 to 15 %E, 15 to 20 %E, >20 %E)	Age; sex; BMI; nonalcoholic energy intake; nutrient density
			Malmö Diet and Cancer Study	---		
MacIntyre et al., 2012 (13)	Added sugars (%E)	Vitamin D (µg/4.18 MJ)	Males	0 (all pairwise comparisons)	Multivariate nonparametric tests for vitamin D intake between quartiles of sugar intake	None; sex; all healthy; nutrient density
			Females	- (Q1 v Q3)<br>0 (all other comparisons)		
	Added sugars (g)	Vitamin D (µg)	Males	+ (Q1 v Q4)<br>0 (all other comparisons)		None; sex; all healthy
			Females	0 (all pairwise comparisons)		
Maunder et al., 2015 (14)	Added sugars (g)	Vitamin D (µg)		+++	Pearson's correlations for vitamin D intake and sugar intake	None; age group (4–8 y)
				0	Pearson's partial correlations for vitamin D intake and sugar intake	Energy intake; age group (4–8 y)
	Added sugars (g/4.18 MJ)	Vitamin D (µg/4.18 MJ)		0	Pearson's correlations for vitamin D intake and sugar intake	None; age group (4–8 y); nutrient density
	Added sugars (%E)	Vitamin D (µg/4.18 MJ)		+ (Q1 v Q2)[4]<br>+ (Q1 v Q3)[4]<br>+ (Q1 v Q4)[4]<br>0 (all other comparisons)	Bonferroni comparisons for vitamin D intake between quartiles of sugar intake	
Morales-Suarez-Varela et al., 2020 (18)	Simple sugars (g)	Vitamin D (µg)	Males	+++	ANOVA for vitamin D intake across three groups of sugar intake (<25th percentile, 25th–75th percentile, >75th percentile)	None; age group (6–8 y); sex; all healthy
			Females	+++		

Study, year (ref)	Sugar type (unit)	Nutrient (unit)	Subgroup	Association reported[1]	Analysis for measures of effect	Confounders accounted for in analysis; in design
	Total sugars (%E)	Vitamin D (µg/1,000 kcal)	Males	0		None; age group (6–8 y); sex; all healthy; nutrient density
			Females	0		
Moshtaghian et al., 2016 (19)	Added sugars (%E)	Vitamin D (µg)		---	Linear regression for vitamin D intake across three sugar intake categories	Age; sex; energy; BMI; nutrient density
				0 (<5 v 5 to 10 %E)<br>- (<5 v >10 %E)<br>0 (5 to 10 v >10 %E)	ANCOVA for vitamin D intake between three sugar intake categories (<5 %E, 5 to 10 %E, >10 %E)	
Wang et al., 2015 (23)	Added sugars (g)	Vitamin D (µg/1,000 kcal)		0 (solid foods)<br>- (liquid foods)	ANOVA for vitamin D intake across tertiles of sugar intake	None; age group (8–10 y); nutrient density
				Liquid foods:<br>0 (T1 v T2)<br>- (T1 v T3)<br>0 (T2 v T3)	Post hoc multiple comparisons (using Duncan and Dunnett's T3 for equal and unequal variances, respectively) for vitamin D intake between tertiles of sugar intake	
Wang et al., 2020 (24)	Total sugars (%E)	Vitamin D (µg/1,000 kcal)		+++[3], non-linear	General linear model for vitamin D intake across quintiles of sugar intake	Age; sex; smoking; self-perceived health; blood pressure; diabetes; heart disease; cancer; osteoporosis; education; physical activity; income; BMI; immigrant status; weekend reference day; misreporting status; nutrient density
				+ (Q1 v Q3)<br>+ (Q1 v Q5)<br>0 (Q3 v Q5)	Post hoc comparisons with Bonferroni adjustments for vitamin D intake between quintiles of sugar intake (Q2 and Q4 NR)	
			Males (19–30 y)	0	General linear model for vitamin D intake across quintiles of sugar intake	Age; sex; smoking; self-perceived health; blood pressure; diabetes; heart disease; cancer; osteoporosis; education; physical activity; income; BMI; immigrant status; weekend reference day; age group; misreporting status; nutrient density
			Males (31–50 y)	0		
			Males (51–70 y)	0		

Study, year (ref)	Sugar type (unit)	Nutrient (unit)	Subgroup	Association reported[1]	Analysis for measures of effect	Confounders accounted for in analysis; in design
			Males (71+ y)	0		
			Females (19–30 y)	0		
			Females (31–50 y)	0		
			Females (51–70 y)	0		
			Females (71+ y)	0		
**Calcium**						
Chiavaroli et al., 2022 (2)	Total sugars (%E)	Calcium (mg/1,000 kcal)	2–8 y	++	General linear model for calcium intake across quintiles of sugar intake	Misreporting status; sex; immigrant status; weekend reference day; age group; nutrient density
				0 (Q1 v Q3)<br>0 (Q1 v Q5)<br>0 (Q3 v Q5)	Bonferroni for calcium intake between quintiles of sugar intake (Q2 and Q4 NR)	
			9–13 y	0	General linear model for calcium intake across quintiles of sugar intake	
			14–18 y	+++		
				+ (Q1 v Q3)<br>+ (Q1 v Q5)<br>0 (Q3 v Q5)	Bonferroni for calcium intake between quintiles of sugar intake (Q2 and Q4 NR)	
Fujiwara et al., 2020 (3)	Free sugars (%E)	Calcium (mg)		0	Linear regression for calcium intake across four sugar intake groups	Age; sex, weight status; age group (1–19 y); nutrient density
				+++ (<2.5 v 2.5 to <5 %E)<br>+++ (<2.5 v 5 to <10 %E)<br>--- (5 to <10 v ≥10 %E)<br>0 (all other comparisons)	Tukey-Kramer test for calcium intake between four sugar intake groups (<2.5 %E, 2.5 to <5 %E, 5 to <10 %E, ≥10 %E)	
		Calcium (mg/1,000 kcal)		-[3], non-linear	Linear regression for calcium intake across four sugar intake groups	
				--- (2.5 to <5 v ≥10 %E)<br>--- (5 to <10 v ≥10 %E)<br>0 (all other comparisons)	Tukey-Kramer test for calcium intake between four sugar intake groups (<2.5 %E, 2.5 to <5 %E, 5 to <10 %E, ≥10 %E)	
Fulgoni et al., 2019 (4)	Added sugars (%E)	Calcium (percentage of population below the EAR)	2–8 y	Sugar intake decile was positively associated with the percentage of the population	Linear regression for the percentage of the population below the EAR for calcium across deciles of sugar intake	Energy intake; age group; nutrient density

Study, year (ref)	Sugar type (unit)	Nutrient (unit)	Subgroup	Association reported[1]	Analysis for measures of effect	Confounders accounted for in analysis; in design
				below the EAR for calcium (*P* = 0.0007)		
			9–18 y	Sugar intake decile was positively associated with the percentage of the population below the EAR for calcium (*P* < 0.0001)		
Fulgoni et al., 2020 (5)	Added sugars (%E)	Calcium (percentage of population below the EAR)	2–18 y	Sugar intake decile was positively associated with the percentage of the population below the EAR for calcium, regardless of the method used (*P*-range: 0.0027 to 0.0001)	Regression for percentage of the population below the EAR for calcium across deciles of sugar intake by various methods (1 day intake, 2 day average intake, UI/NCI, and UI/MCMC)	None; age group; nutrient density
			≥19 y	Sugar intake decile was not associated with the percentage of population below the EAR for calcium for all methods (*P* > 0.2) except UI/MCMC, which showed a negative association (*P* = 0.0012)		
Gibson et al., 2016 (6)	Free sugars (%E)[2]	Calcium (mg/4.18 MJ)	4–10 y	---	ANOVA for calcium intake across quintiles of sugar intake	None; age group; nutrient density
				- (Q1 v Q5)<br>- (Q2 v Q5)<br>0 (all other comparisons)	Bonferroni comparisons of calcium intake between quintiles of sugar intake	
			11–18 y	--	ANOVA for calcium intake across quintiles of sugar intake	
				0 (all pairwise comparisons)	Bonferroni comparisons of calcium intake between quintiles of sugar intake	
		Calcium (mg)	4–10 y	0	ANOVA for calcium intake across quintiles of sugar intake	
			11–18 y	0	ANOVA for calcium intake across quintiles of sugar intake	
Goletzke et al., 2015 (7)	Total sugars (g/1,000 kJ)	Calcium (mg)		+	Cross-sectional estimate: linear mixed regression model for increase/decrease in calcium intake at baseline associated with a 1 g/1,000 kJ increase in sugar intake	Age at study entry; prepregnancy BMI; ethnicity; intervention group; energy; sex (all female); all healthy; nutrient density

Study, year (ref)	Sugar type (unit)	Nutrient (unit)	Subgroup	Association reported[1]	Analysis for measures of effect	Confounders accounted for in analysis; in design
				++	20 wk concurrent change estimate: linear mixed regression model for change in calcium intake associated with a 1 g/1,000 kJ increase in sugar intake over a mean 20 wk of gestation	
González-Padilla et al., 2020 (10)	Added sugars (%E)	Calcium (mg)	Riksmaten Adults	---	General linear model for calcium intake across six groups of sugar intake (<5 %E, 5 to 7.5 %E, 7.5 to 10 %E, 10 to 15 %E, 15 to 20 %E, >20 %E)	Age; sex; BMI; nonalcoholic energy intake; nutrient density
			Malmö Diet and Cancer Study	---		
MacIntyre et al., 2012 (13)	Added sugars (%E)	Calcium (mg/4.18 MJ)	Males	0 (all pairwise comparisons)	Multivariate nonparametric tests for calcium intake across quartiles of sugar intake	None; sex; all healthy; nutrient density
			Females	- (Q1 v Q4)<br>0 (all other comparisons)		
	Added sugars (g)	Calcium (mg)	Males	+ (Q1 v Q4)<br>0 (all other comparisons)		None; sex; all healthy
			Females	+ (Q1 v Q4)<br>+ (Q2 v Q4)<br>0 (all other comparisons)		
Maunder et al., 2015 (14)	Added sugars (g)	Calcium (mg)		+++	Pearson's correlations for calcium intake and sugar intake	None; age group (4–8 y)
				0	Pearson's partial correlations for calcium intake and sugar intake	Energy intake; age group (4–8 y)
	Added sugars (g/4.18 MJ)	Calcium (mg/4.18 MJ)		0	Pearson's correlations for calcium intake and sugar intake	None; age group (4–8 y), nutrient density
	Added sugars (%E)	Calcium (mg/4.18 MJ)		0 (all pairwise comparisons)	Bonferroni comparisons for calcium intake between quartiles of sugar intake	
Morales-Suarez-Varela et al., 2020 (18)	Simple sugars (g)	Calcium (mg)	Males	+++	ANOVA for calcium intake across three groups of sugar intake (<25th percentile, 25th–75th percentile, >75th percentile)	None; age group (6–8 y), sex, all healthy
			Females	+++		

Study, year (ref)	Sugar type (unit)	Nutrient (unit)	Subgroup	Association reported[1]	Analysis for measures of effect	Confounders accounted for in analysis; in design
	Total sugars (%E)	Calcium (mg/1,000 kcal)	Males	+++		None; age group (6–8 y), sex, all healthy, nutrient density
			Females	+++		
Moshtaghian et al., 2016 (19)	Added sugars (%E)	Calcium (mg)		---	Linear regression for calcium intake across three sugar intake categories	Age; sex; energy: BMI; nutrient density
				0 (<5 v 5 to 10 %E)<br>- (<5 v >10 %E)<br>- (5 to 10 v >10 %E)	ANCOVA for calcium intake across three sugar intake categories (<5 %E, 5 to 10 %E, >10 %E)	
Wang et al., 2015 (23)	Added sugars (g)	Calcium (mg/1,000 kcal)		0 (solid foods)<br>--- (liquid foods)	ANOVA for calcium intake across tertiles of sugar intake	None; age group (8–10 y); nutrient density
				Solid foods:<br>0 (T1 v T2)<br>- (T1 v T3)<br>0 (T2 v T3)<br>Liquid foods:<br>- (T1 v T2)<br>- (T1 v T3)<br>0 (T2 v T3)	Post hoc multiple comparisons (using Duncan and Dunnett's T3 for equal and unequal variances, respectively) for calcium intake between tertiles of sugar intake	
Wang et al., 2020 (24)	Total sugars (%E)	Calcium (mg/1,000 kcal)		+++	General linear model for calcium intake across quintiles of sugar intake	Age; sex; smoking; self-perceived health; blood pressure; diabetes; heart disease; cancer; osteoporosis; education; physical activity; income; BMI; immigrant status; weekend reference day; misreporting status; nutrient density
				0 (Q1 v Q3)<br>0 (Q1 v Q5)<br>0 (Q3 v Q5)	Post hoc comparisons with Bonferroni adjustments for calcium intake between quintiles of sugar intake (Q2 and Q4 NR)	
			Males (19–30 y)	0	General linear model for calcium intake across quintiles of sugar intake	Age; sex; smoking; self-perceived health; blood pressure; diabetes; heart disease; cancer; osteoporosis; education; physical activity; income; BMI; immigrant status; weekend reference day; misreporting status; age group; nutrient density

Study, year (ref)	Sugar type (unit)	Nutrient (unit)	Subgroup	Association reported[1]	Analysis for measures of effect	Confounders accounted for in analysis; in design
			Males (31–50 y)	0		
			Males (51–70 y)	+++		
				+ (Q1 v Q3)<br>+ (Q1 v Q5)<br>0 (Q3 v Q5)	Post hoc comparisons with Bonferroni adjustments for calcium intake between quintiles of sugar intake (Q2 and Q4 NR)	
			Males (71+ y)	+++	General linear model for calcium intake across quintiles of sugar intake	
				+ (Q1 v Q3)<br>+ (Q1 v Q5)<br>0 (Q3 v Q5)	Post hoc comparisons with Bonferroni adjustments for calcium intake between quintiles of sugar intake (Q2 and Q4 NR)	
			Females (19–30 y)	+	General linear model for calcium intake across quintiles of sugar intake	
				0 (Q1 v Q3)<br>0 (Q1 v Q5)<br>0 (Q3 v Q5)	Post hoc comparisons with Bonferroni adjustments for calcium intake between quintiles of sugar intake (Q2 and Q4 NR)	
			Females (31–50 y)	+++	General linear model for calcium intake across quintiles of sugar intake	
				+ (Q1 v Q3)<br>0 (Q1 v Q5)<br>0 (Q3 v Q5)	Post hoc comparisons with Bonferroni adjustments for calcium intake between quintiles of sugar intake (Q2 and Q4 NR)	
			Females (51–70 y)	+++	General linear model for calcium intake across quintiles of sugar intake	
				0 (Q1 v Q3)<br>0 (Q1 v Q5)<br>0 (Q3 v Q5)	Post hoc comparisons with Bonferroni adjustments for calcium intake between quintiles of sugar intake (Q2 and Q4 NR)	
			Females (71+ y)	++	General linear model for calcium intake across quintiles of sugar intake	
				0 (Q1 v Q3)<br>0 (Q1 v Q5)<br>0 (Q3 v Q5)	Post hoc comparisons with Bonferroni adjustments for calcium intake between quintiles of sugar intake (Q2 and Q4 NR)	
Wong et al., 2019 (26)	Free sugars (%E)	Calcium (mg)		---[3], non-linear	Linear regression for calcium intake across six free sugar cut-off levels (<5 %E, 5 to <10 %E, 10 to <15 %E, 15 to <20 %E, 20 to 25 %E, >25 %E)	Age; sex; socio-economic indices of disadvantage for areas (SEIFA); equivalized household income; remoteness of living area; age group (2–18 y); nutrient density

Study, year (ref)	Sugar type (unit)	Nutrient (unit)	Subgroup	Association reported[1]	Analysis for measures of effect	Confounders accounted for in analysis; in design
		Calcium (mg/1,000 kJ)		---		
**Potassium**						
Chiavaroli et al., 2022 (2)	Total sugars (%E)	Potassium (mg/1,000 kcal)	2–8 y	+++	General linear model for potassium intake across quintiles of sugar intake	Misreporting status; sex; immigrant status; weekend reference day; age group; nutrient density
				+ (Q1 v Q3)<br>+ (Q1 v Q5)<br>0 (Q3 v Q5)	Post hoc comparisons with Bonferroni corrections for potassium intake between quintiles of sugar intake (Q2 and Q4 NR)	
			9–13 y	+	General linear model for potassium intake across quintiles of sugar intake	
				0 (Q1 v Q3)<br>0 (Q1 v Q5)<br>0 (Q3 v Q5)	Post hoc comparisons with Bonferroni corrections for potassium intake between quintiles of sugar intake (Q2 and Q4 NR)	
			14–18 y	+++	General linear model for potassium intake across quintiles of sugar intake	
				0 (Q1 v Q3)<br>+ (Q1 v Q5)<br>0 (Q3 v Q5)	Post hoc comparisons with Bonferroni corrections for potassium intake between quintiles of sugar intake (Q2 and Q4 NR)	
Fujiwara et al., 2020 (3)	Free sugars (%E)	Potassium (mg)		--[3], non-linear	Linear regression for potassium intake across four sugar intake levels	Age; sex; weight status; age group (1–19 y); nutrient density
				+++ (<2.5 v 2.5 to <5 %E)<br>+++ (<2.5 v 5 to <10 %E)<br>--- (2.5 to <5 v ≥10 %E)<br>--- (5 to <10 v ≥10 %E)<br>0 (all other comparisons)	Tukey-Kramer test for potassium intake between sugar intake levels (<2.5 %E, 2.5 to <5 %E, 5 to <10 %E, ≥10 %E)	
		Potassium (mg/1,000 kcal)		---[3], non-linear	Linear regression for potassium intake across four sugar intake levels	
				--- (<2.5 v ≥10 %E)<br>--- (2.5 to <5 v 5 to <10 %E)<br>--- (2.5 to <5 v ≥10 %E)<br>--- (5 to <10 v ≥10 %E)<br>0 (all other comparisons)	Tukey-Kramer test for potassium intake between sugar intake levels (<2.5 %E, 2.5 to <5 %E, 5 to <10 %E, ≥10 %E)	
Fulgoni et al., 2020 (5)	Added sugars (%E)	Potassium (percentage of population above the AI)	2–18 y	Sugar intake decile was negatively associated with the percentage of the population above the AI for potassium, regardless of the	Regression for potassium intake across deciles of sugar intake by various methods (1 day intake, 2 day average intake, UI/NCI, and UI/MCMC)	None; age group; nutrient density

Study, year (ref)	Sugar type (unit)	Nutrient (unit)	Subgroup	Association reported[1]	Analysis for measures of effect	Confounders accounted for in analysis; in design
				method used (*P*-range: 0.007 to < 0.0001)		
			≥19 y	Sugar intake decile was negatively associated with the percentage of the population above the AI for potassium, regardless of the method used (*P*-range: 0.005 to 0.0002)		
Gibson et al., 2016 (6)	Free sugars (%E)[2]	Potassium (mg/4.18 MJ)	4–10 y	--[3], non-linear	ANOVA for potassium intake across quintiles of sugar intake	None; age group; nutrient density
				- (Q1 v Q5)<br>0 (all other comparisons)	Bonferroni comparisons for potassium intake between quintiles of sugar intake	
			11–18 y	---	ANOVA for potassium intake across quintiles of sugar intake	
				- (Q1 v Q3)<br>- (Q1 v Q4)<br>- (Q1 v Q5)<br>0 (all other comparisons)	Bonferroni comparisons for potassium intake between quintiles of sugar intake	
		Potassium (mg)	4–10 y	0	ANOVA for potassium intake across quintiles of sugar intake	
			11–18 y	0	ANOVA for potassium intake across quintiles of sugar intake	
Goletzke et al., 2015 (7)	Total sugars (g/1,000 kJ)	Potassium (mg)		+++	Cross-sectional estimate: linear mixed regression model for increase/decrease in potassium intake at baseline associated with a 1 g/1,000 kJ increase in sugar intake	Age at study entry; prepregnancy BMI; ethnicity; intervention group; energy; sex (all female); all healthy; nutrient density
				+++	20 wk concurrent change estimate: linear mixed regression model for change in potassium intake associated with a 1 g/1,000 kJ increase in sugar intake over a mean 20 wk of gestation	
González-Padilla et al., 2020 (10)	Added sugars (%E)	Potassium (mg)	Riksmaten Adults	---	General linear model for potassium intake across six groups of sugar intake (<5 %E, 5 to 7.5 %E, 7.5 to 10 %E, 10 to 15 %E, 15 to 20 %E, >20 %E)	Age; sex; BMI; nonalcoholic energy intake; nutrient density

Study, year (ref)	Sugar type (unit)	Nutrient (unit)	Subgroup	Association reported[1]	Analysis for measures of effect	Confounders accounted for in analysis; in design
			Malmö Diet and Cancer Study	---		
Wang et al., 2020 (24)	Total sugars (%E)	Potassium (mg/1,000 kcal)		+++	General linear model for potassium intake across quintiles of sugar intake	Age; sex; smoking; self-perceived health; blood pressure; diabetes; heart disease; cancer; osteoporosis; education; physical activity; income; BMI; immigrant status; weekend reference day; misreporting status; nutrient density
				0 (Q1 v Q3)<br>0 (Q1 v Q5)<br>0 (Q3 v Q5)	Post hoc comparisons with Bonferroni adjustments for potassium intake between quintiles of sugar intake (Q2 and Q4 NR)	
			Males (19–30 y)	++	General linear model for potassium intake across quintiles of sugar intake	Age; sex; smoking; self-perceived health; blood pressure; diabetes; heart disease; cancer; osteoporosis; education; physical activity; income; BMI; immigrant status; weekend reference day; misreporting status; age group; nutrient density
				0 (Q1 v Q3)<br>+ (Q1 v Q5)<br>0 (Q3 v Q5)	Post hoc comparisons with Bonferroni adjustments for potassium intake between quintiles of sugar intake (Q2 and Q4 NR)	
			Males (31–50 y)	0	General linear model for potassium intake across quintiles of sugar intake	
			Males (51–70 y)	++		
				+ (Q1 v Q3)<br>+ (Q1 v Q5)<br>0 (Q3 v Q5)	Post hoc comparisons with Bonferroni adjustments for potassium intake between quintiles of sugar intake (Q2 and Q4 NR)	
			Males (71+ y)	+++	General linear model for potassium intake across quintiles of sugar intake	
				+ (Q1 v Q3)<br>+ (Q1 v Q5)<br>0 (Q3 v Q5)	Post hoc comparisons with Bonferroni adjustments for potassium intake between quintiles of sugar intake (Q2 and Q4 NR)	

Study, year (ref)	Sugar type (unit)	Nutrient (unit)	Subgroup	Association reported[1]	Analysis for measures of effect	Confounders accounted for in analysis; in design
			Females (19–30 y)	0	General linear model for potassium intake across quintiles of sugar intake	
			Females (31–50 y)	+++		
				+ (Q1 v Q3)<br>+ (Q1 v Q5)<br>0 (Q3 v Q5)	Post hoc comparisons with Bonferroni adjustments for potassium intake between quintiles of sugar intake (Q2 and Q4 NR)	
			Females (51–70 y)	+	General linear model for potassium intake across quintiles of sugar intake	
				0 (Q1 v Q3)<br>0 (Q1 v Q5)<br>0 (Q3 v Q5)	Post hoc comparisons with Bonferroni adjustments for potassium intake between quintiles of sugar intake (Q2 and Q4 NR)	
			Females (71+ y)	++	General linear model for potassium intake across quintiles of sugar intake	
				0 (Q1 v Q3)<br>+ (Q1 v Q5)<br>0 (Q3 v Q5)	Post hoc comparisons with Bonferroni adjustments for potassium intake between quintiles of sugar intake (Q2 and Q4 NR)	
**Sodium**						
Chiavaroli et al., 2022 (2)	Total sugars (%E)	Sodium (mg/1,000 kcal)	2–8 y	---	General linear model for sodium intake across quintiles of sugar intake	Misreporting status; sex; immigrant status; weekend reference day; age group; nutrient density
				0 (Q1 v Q3)<br>- (Q1 v Q5)<br>- (Q3 v Q5)	Post hoc comparisons with Bonferroni corrections for sodium intake between quintiles of sugar intake (Q2 and Q4 NR)	
			9–13 y	---	General linear model for sodium intake across quintiles of sugar intake	
				0 (Q1 v Q3)<br>- (Q1 v Q5)<br>0 (Q3 v Q5)	Post hoc comparisons with Bonferroni corrections for sodium intake between quintiles of sugar intake (Q2 and Q4 NR)	
			14–18 y	---	General linear model for sodium intake across quintiles of sugar intake	
				- (Q1 v Q3)<br>- (Q1 v Q5)<br>0 (Q3 v Q5)	Post hoc comparisons with Bonferroni corrections for sodium intake between quintiles of sugar intake (Q2 and Q4 NR)	
Fujiwara et al., 2020 (3)	Free sugars (%E)	Sodium (mg)		0	Linear regression for sodium intake across four sugar intake levels	Age; sex; weight status; age group (1–19 y); nutrient density

Study, year (ref)	Sugar type (unit)	Nutrient (unit)	Subgroup	Association reported[1]	Analysis for measures of effect	Confounders accounted for in analysis; in design
				+++ (<2.5 v 2.5 to <5 %E)<br>+++ (<2.5 v 5 to <10 %E)<br>0 (all other comparisons)	Tukey-Kramer test for sodium intake between sugar intake levels (<2.5 %E, 2.5 to <5 %E, 5 to <10 %E, ≥10 %E)	
		Sodium (mg/1,000 kcal)		---[3], non-linear	Linear regression for sodium intake across four sugar intake levels	
				--- (2.5 to <5 v ≥10 %E)<br>0 (all other comparisons)	Tukey-Kramer test for sodium intake between sugar intake levels (<2.5 %E, 2.5 to <5 %E, 5 to <10 %E, ≥10 %E)	
Goletzke et al., 2015 (7)	Total sugars (g/1,000 kJ)	Sodium (mg)		-	Cross-sectional estimate: linear mixed regression model for increase/decrease in sodium intake at baseline associated with a 1 g/1,000 kJ increase in sugar intake	Age at study entry; prepregnancy BMI; ethnicity; intervention group; energy; sex (all female); all healthy; nutrient density
				--	20 wk concurrent change estimate: linear mixed regression model for change in sodium intake associated with a 1 g/1,000 kJ increase in sugar intake over a mean 20 wk of gestation	
Gress et al., 2020 (11)	Total sugars (g)	Sodium (mg)		---	Linear regression for sugar intake across quintiles of sodium intake	None
				--- (Q1 v Q3)[5]<br>--- (Q2 v Q3)[5]<br>--- (Q4 v Q3)[5]<br>--- (Q5 v Q3)[5]	Multiple linear regression for sugar intake across quintiles of sodium intake; each quintile was compared to quintile 3 (reference)	Age; sex; BMI, physical activity; smoking; hypertension; diabetes; coronary artery disease; congestive heart failure; glycosylated hemoglobin; season[5]
Sluik et al., 2016 (21)	Added sugars (%E)	Sodium (component score, 0-10)	Males	0 (<10 v ≥10 %E)<br>0 (<20 v ≥20 %E)	Independent t-test for sodium intake between guidelines adherent ("<" %E from sugar) and non-adherent ("≥" %E from sugar) groups	None; age group (19-69 y); sex; nutrient density
			Females	0 (<10 v ≥10 %E)<br>0 (<20 v ≥20 %E)		
	Free sugars (%E)		Males	0 (<5 v ≥5 %E)<br>0 (<10 v ≥10 %E)		
			Females	0 (<5 v ≥5 %E)<br>0 (<10 v ≥10 %E)		
Wang et al., 2020 (24)	Total sugars (%E)	Sodium (mg/1,000 kcal)		---	General linear model for sodium intake across quintiles of sugar intake	Age; sex; smoking; self-perceived health; blood pressure; diabetes; heart disease; cancer; osteoporosis; education; physical activity;

Study, year (ref)	Sugar type (unit)	Nutrient (unit)	Subgroup	Association reported[1]	Analysis for measures of effect	Confounders accounted for in analysis; in design
						income; BMI; immigrant status; weekend reference day; misreporting status; nutrient density
				0 (Q1 v Q3)<br>0 (Q1 v Q5)<br>- (Q3 v Q5)	Post hoc comparisons with Bonferroni adjustments for sodium intake between quintiles of sugar intake (Q2 and Q4 NR)	
			Males (19–30 y)	---	General linear model for sodium intake across quintiles of sugar intake	Age; sex; smoking; self-perceived health; blood pressure; diabetes; heart disease; cancer; osteoporosis; education; physical activity; income; BMI; immigrant status; weekend reference day; misreporting status; age group; nutrient density
				0 (Q1 v Q3)<br>- (Q1 v Q5)<br>- (Q3 v Q5)	Post hoc comparisons with Bonferroni adjustments for sodium intake between quintiles of sugar intake (Q2 and Q4 NR)	
			Males (31–50 y)	--	General linear model for sodium intake across quintiles of sugar intake	
				0 (Q1 v Q3)<br>- (Q1 v Q5)<br>- (Q3 v Q5)	Post hoc comparisons with Bonferroni adjustments for sodium intake between quintiles of sugar intake (Q2 and Q4 NR)	
			Males (51–70 y)	--	General linear model for sodium intake across quintiles of sugar intake	
				0 (Q1 v Q3)<br>- (Q1 v Q5)<br>0 (Q3 v Q5)	Post hoc comparisons with Bonferroni adjustments for sodium intake between quintiles of sugar intake (Q2 and Q4 NR)	
			Males (71+ y)	---	General linear model for sodium intake across quintiles of sugar intake	
				0 (Q1 v Q3)<br>- (Q1 v Q5)<br>0 (Q3 v Q5)	Post hoc comparisons with Bonferroni adjustments for sodium intake between quintiles of sugar intake (Q2 and Q4 NR)	
			Females (19–30 y)	-	General linear model for sodium intake across quintiles of sugar intake	
				0 (Q1 v Q3)<br>0 (Q1 v Q5)<br>0 (Q3 v Q5)	Post hoc comparisons with Bonferroni adjustments for sodium intake between quintiles of sugar intake (Q2 and Q4 NR)	

Study, year (ref)	Sugar type (unit)	Nutrient (unit)	Subgroup	Association reported[1]	Analysis for measures of effect	Confounders accounted for in analysis; in design
			Females (31–50 y)	---	General linear model for sodium intake across quintiles of sugar intake	
				0 (Q1 v Q3)<br>- (Q1 v Q5)<br>- (Q3 v Q5)	Post hoc comparisons with Bonferroni adjustments for sodium intake between quintiles of sugar intake (Q2 and Q4 NR)	
			Females (51–70 y)	---	General linear model for sodium intake across quintiles of sugar intake	
				0 (Q1 v Q3)<br>- (Q1 v Q5)<br>- (Q3 v Q5)	Post hoc comparisons with Bonferroni adjustments for sodium intake between quintiles of sugar intake (Q2 and Q4 NR)	
			Females (71+ y)	--	General linear model for sodium intake across quintiles of sugar intake	
				0 (Q1 v Q3)<br>- (Q1 v Q5)<br>0 (Q3 v Q5)	Post hoc comparisons with Bonferroni adjustments for sodium intake between quintiles of sugar intake (Q2 and Q4 NR)	
Wong et al., 2019 (26)	Free sugars (%E)	Sodium (mg)		---[3], non-linear	Linear regression for sodium intake across six free sugar cut-off levels (<5 %E, 5 to <10 %E, 10 to <15 %E, 15 to <20 %E, 20 to 25 %E, >25 %E)	Age; sex; socio-economic indices of disadvantage for areas (SEIFA); equivalized household income; remoteness of living area; age group (2–18 y); nutrient density
		Sodium (mg/1,000 kJ)		---[3], non-linear		

%E, percent of total energy intake; AI, adequate intake; ANCOVA, analysis of covariance; ANOVA, analysis of variance; BMI, body mass index; d, day(s); EAR, estimated average requirement; g, grams; MCMC, Markov Chain Monte Carlo; NCI, National Cancer Institute; NR, not reported; Q, quantile; T, tertile; UI, usual intake; v, versus; wk, week(s); y, years

[1] Associations reported as: significant negative association (-, $P \leq 0.05$; --, $P \leq 0.01$; ---, $P \leq 0.001$); no association (0, $P > 0.05$); significant positive association (+, $P \leq 0.05$; ++, $P \leq 0.01$; +++, $P \leq 0.001$).
[2] Reported using non-milk extrinsic sugars as a proxy for free sugar.
[3] The trend across groups was not linear, so the association reported represents the comparison between the lowest and highest sugar intake groups.
[4] Authors did not specify *P*-levels for significant pairwise comparisons reported in the text; $P < 0.05$ was presumed based on the Bonferroni significance levels reported in Table 5 (14).
[5] The same conclusions and significance levels were reported when the models serially excluded cohort members with CVD, hypertension, and diabetes.

Supplemental References

1. Castellanos-Gutierrez A, Rodriguez-Ramirez S, Bromage S, Fung TT, Li Y, Bhupathiraju SN, Deitchler M, Willett W, Batis C. Performance of the Global Diet Quality Score with Nutrition and Health Outcomes in Mexico with 24-h Recall and FFQ Data. J Nutr 2021;151(12 Suppl 2):143S-51S. doi: 10.1093/jn/nxab202.

2. Chiavaroli L, Wang YF, Ahmed M, Ng AP, DiAngelo C, Marsden S, Sievenpiper JL. Intakes of nutrients and food categories in Canadian children and adolescents across levels of sugars intake: cross-sectional analyses of the Canadian Community Health Survey 2015 Public Use Microdata File. Appl Physiol Nutr Metab 2022;47(4):415-28. doi: 10.1139/apnm-2021-0517.

3. Fujiwara A, Okada E, Okada C, Matsumoto M, Takimoto H. Association between free sugars intake and nutrient dilution among Japanese adults: the 2016 National Health and Nutrition Survey, Japan. Eur J Nutr 2020;59(8):3827-39. doi: 10.1007/s00394-020-02213-4.

4. Fulgoni VL, Gaine PC, Scott MO, Ricciuto L, Difrancesco L. Association of added sugars intake with micronutrient adequacy in US children and adolescents: NHANES 2009-2014. Current Developments in Nutrition 2019;3(12):nzz126.

5. Fulgoni VL, 3rd, Gaine PC, Scott MO. Comparison of Various Methods to Determine Added Sugars Intake to Assess the Association of Added Sugars Intake and Micronutrient Adequacy. Nutrients 2020;12(9). doi: https://dx.doi.org/10.3390/nu12092816.

6. Gibson S, Francis L, Newens K, Livingstone B. Associations between free sugars and nutrient intakes among children and adolescents in the UK. Br J Nutr 2016;116(7):1265-74. doi: 10.1017/S0007114516003184.

7. Goletzke J, Buyken AE, Louie JC, Moses RG, Brand-Miller JC. Dietary micronutrient intake during pregnancy is a function of carbohydrate quality. Am J Clin Nutr 2015;102(3):626-32. doi: 10.3945/ajcn.114.104836.

8. Golley RK, Hendrie GA, McNaughton SA. Scores on the dietary guideline index for children and adolescents are associated with nutrient intake and socio-economic position but not adiposity. J Nutr 2011;141(7):1340-7. doi: 10.3945/jn.110.136879.

9. Gomez G, Nogueira Previdelli A, Fisberg RM, Kovalskys I, Fisberg M, Herrera-Cuenca M, Cortes Sanabria LY, Yepez Garcia MC, Rigotti A, Liria-Dominguez MR, et al. Dietary Diversity and Micronutrients Adequacy in Women of Childbearing Age: Results from ELANS Study. Nutrients 2020;12(7). doi: 10.3390/nu12071994.

10. Gonzalez-Padilla E, J AD, Ramne S, Olsson K, Nalsen C, Sonestedt E. Association between added sugar intake and micronutrient dilution: a cross-sectional study in two adult Swedish populations. Nutr Metab (Lond) 2020;17(1):15. doi: 10.1186/s12986-020-0428-6.

11. Gress TW, Mansoor K, Rayyan YM, Khthir RA, Tayyem RF, Tzamaloukas AH, Abraham NG, Shapiro JI, Khitan ZJ. Relationship between dietary sodium and sugar intake: A cross-sectional study of the National Health and Nutrition Examination Survey 2001-2016. J Clin Hypertens (Greenwich) 2020;22(9):1694-702. doi: 10.1111/jch.13985.

12. Kaartinen NE, Simila ME, Kanerva N, Valsta LM, Harald K, Mannisto S. Naturally occurring and added sugar in relation to macronutrient intake and food consumption: results from a population-based study in adults. J Nutr Sci 2017;6:e7. doi: 10.1017/jns.2017.3.

13. MacIntyre U, Venter C, Kruger A, Serfontein M. Measuring micronutrient intakes at different levels of sugar consumption in a population in transition: the Transition and Health during Urbanisation in South Africa (THUSA) study. South African Journal of Clinical Nutrition 2012;25(3):122-30. doi: 10.1080/16070658.2012.11734416.

14. Maunder EM, Nel JH, Steyn NP, Kruger HS, Labadarios D. Added Sugar, Macro- and Micronutrient Intakes and Anthropometry of Children in a Developing World Context. PLoS One 2015;10(11):e0142059. doi: 10.1371/journal.pone.0142059.

15. Meira RCF, Capitani CD, Barros Filho AA, Barros MBA, Assumpcao D. Contribution of different foods according to the Nova classification to dietary fiber intake in adolescents. Cien Saude Colet 2021;26(8):3147-60. doi: 10.1590/1413-81232021268.09592020.

16. Montejano Vallejo R, Schulz CA, van de Locht K, Oluwagbemigun K, Alexy U, Nothlings U. Associations of Adherence to a Dietary Index Based on the EAT-Lancet Reference Diet with Nutritional, Anthropometric, and Ecological Sustainability Parameters: Results from the German DONALD Cohort Study. J Nutr 2022;152(7):1763-72. doi: 10.1093/jn/nxac094.

17. Moraeus L, Lindroos AK, Warensjo Lemming E, Mattisson I. Diet diversity score and healthy eating index in relation to diet quality and socio-demographic factors: results from a cross-sectional national dietary survey of Swedish adolescents. Public Health Nutr 2020;23(10):1754-65. doi: 10.1017/S1368980019004671.

18. Morales-Suarez-Varela M, Peraita-Costa I, Llopis-Morales A, Pico Y, Bes-Rastrollo M, Llopis-Gonzalez A. Total Sugar Intake and Macro and Micronutrients in Children Aged 6-8 Years: The ANIVA Study. Nutrients 2020;12(2). doi: 10.3390/nu12020349.

19. Moshtaghian H, Louie JC, Charlton KE, Probst YC, Gopinath B, Mitchell P, Flood VM. Added sugar intake that exceeds current recommendations is associated with nutrient dilution in older Australians. Nutrition 2016;32(9):937-42. doi: 10.1016/j.nut.2016.02.004.

20. Murakami K. Associations between nutritional quality of meals and snacks assessed by the Food Standards Agency nutrient profiling system and overall diet quality and adiposity measures in British children and adolescents. Nutrition 2018;49:57-65. doi: 10.1016/j.nut.2017.10.011.

21. Sluik D, van Lee L, Engelen AI, Feskens EJ. Total, Free, and Added Sugar Consumption and Adherence to Guidelines: The Dutch National Food Consumption Survey 2007-2010. Nutrients 2016;8(2):70. doi: 10.3390/nu8020070.

22. Voortman T, Kiefte-de Jong JC, Geelen A, Villamor E, Moll HA, Jongste JCd, Raat H, Hofman A, Jaddoe VWV, Franco OH, et al. The development of a diet quality score for preschool children and its validation and determinants in the Generation R Study. Journal of Nutrition 2015;145(2):306-14.

23. Wang J, Shang L, Light K, O'Loughlin J, Paradis G, Gray-Donald K. Associations between added sugar (solid vs. liquid) intakes, diet quality, and adiposity indicators in Canadian children. Appl Physiol Nutr Metab 2015;40(8):835-41. doi: 10.1139/apnm-2014-0447.

24. Wang YF, Chiavaroli L, Roke K, DiAngelo C, Marsden S, Sievenpiper J. Canadian Adults with Moderate Intakes of Total Sugars have Greater Intakes of Fibre and Key Micronutrients: Results from the Canadian Community Health Survey 2015 Public Use Microdata File. Nutrients 2020;12(4). doi: 10.3390/nu12041124.

25. Wong JE, Skidmore PM, Williams SM, Parnell WR. Healthy dietary habits score as an indicator of diet quality in New Zealand adolescents. J Nutr 2014;144(6):937-42. doi: 10.3945/jn.113.188375.

26. Wong THT, Mok A, Ahmad R, Rangan A, Louie JCY. Intake of free sugar and micronutrient dilution in Australian children and adolescents. Eur J Nutr 2019;58(6):2485-95. doi: 10.1007/s00394-018-1801-3.

27. Kelly SE, Greene-Finestone LS, Yetley EA, Benkhedda K, Brooks SPJ, Wells GA, MacFarlane AJ. NUQUEST—NUtrition QUality Evaluation Strengthening Tools: development of tools for the evaluation of risk of bias in nutrition studies. The American Journal of Clinical Nutrition 2021;115(1):256-71. doi: 10.1093/ajcn/nqab335.
